# BACE1 activity regulates cell surface contactin-2 levels

**DOI:** 10.1186/1750-1326-9-4

**Published:** 2014-01-09

**Authors:** Vivek Gautam, Carla D’Avanzo, Matthias Hebisch, Dora M Kovacs, Doo Yeon Kim

**Affiliations:** 1 Genetics and Aging Research Unit, MassGeneral Institute for Neurodegenerative Disease, Massachusetts General Hospital, Harvard Medical School, Charlestown, MA 02129, USA

## Abstract

**Background:**

Although BACE1 is a major therapeutic target for Alzheimer’s disease (AD), potential side effects of BACE1 inhibition are not well characterized. BACE1 cleaves over 60 putative substrates, however the majority of these cleavages have not been characterized. Here we investigated BACE1-mediated cleavage of human contactin-2, a GPI-anchored cell adhesion molecule.

**Results:**

Our initial protein sequence analysis showed that contactin-2 harbors a strong putative BACE1 cleavage site close to its GPI membrane linker domain. When we overexpressed BACE1 in CHO cells stably transfected with human contactin-2, we found increased release of soluble contactin-2 in the conditioned media. Conversely, pharmacological inhibition of BACE1 in CHO cells expressing human contactin-2 and mouse primary neurons decreased soluble contactin-2 secretion. The BACE1 cleavage site mutation 1008MM/AA dramatically impaired soluble contactin-2 release. We then asked whether contactin-2 release induced by BACE1 expression would concomitantly decrease cell surface levels of contactin-2. Using immunofluorescence and surface-biotinylation assays, we showed that BACE1 activity tightly regulates contactin-2 surface levels in CHO cells as well as in mouse primary neurons. Finally, contactin-2 levels were decreased in Alzheimer’s disease brain samples correlating inversely with elevated BACE1 levels in the same samples.

**Conclusion:**

Our results clearly demonstrate that mouse and human contactin-2 are physiological substrates for BACE1. BACE1-mediated contactin-2 cleavage tightly regulates the surface expression of contactin-2 in neuronal cells. Given the role of contactin-2 in cell adhesion, neurite outgrowth and axon guidance, our data suggest that BACE1 may play an important role in these physiological processes by regulating contactin-2 surface levels.

## Introduction

Alzheimer’s disease (AD) is the most common neurodegenerative disease that affects millions of people worldwide. Studies strongly suggest that the accumulation of toxic amyloid -β peptides (Aβ) is associated with synaptic dysfunction and neuronal loss in AD [1]. Aβ peptides are generated from sequential cleavages of the amyloid β precursor protein (APP), which are mediated by the β-site APP cleaving enzyme 1 (BACE1) and Presenilin/γ-secretase [2-4]. Therefore, BACE1 and γ-secretase represent two major therapeutic targets for prevention and treatment of AD.

BACE1, also known as memapsin 2 and Asp 2, is a type I transmembrane aspartyl protease that is highly expressed in neuronal tissues [5,6]. Besides generating pathogenic Aβ, BACE1 also plays crucial roles in numerous physiological processes including neuronal activity, myelination, axonal guidance, presynaptic activity and cognitive behavior in mice [7-14]. These physiological BACE1 functions cast a doubt on the safety of BACE1 inhibition therapy currently being developed to block Aβ generation in AD patients. Currently more than 60 BACE1 substrates have been reported in *in vitro* and *in vivo* conditions [15-21]. Therefore characterizing BACE1-mediated cleavage for each substrate may not only contribute to our understanding of how BACE1 regulates crucial physiological processes but also aid in the prevention of potential side effects deriving from BACE1 inhibition therapy.

Contactin-2 (axonin-1 or transient axonal glycoprotein-1 (TAG-1)) is a cell adhesion molecule that belongs to immunoglobulin super family [22,23]. Contactin-2 is highly expressed at the axon growth cone and plays an important role in regulating axon guidance and path finding [24,25]. Studies from knockout mice revealed that contactin-2 is also crucial for normal learning and memory functions [26]. While the majority of currently reported BACE1 substrates are type I membrane proteins with transmembrane domains, contactin-2 belongs to the carboxy-terminal glycan phosphatidyl inositol (GPI)-anchored protein family that requires covalent linkage of GPI domains for binding to the plasma membrane. Interestingly, two recent unbiased secretome analyses suggested that BACE1 activity regulates the release of contactin-2 in neurons but the BACE1-mediated contactin-2 cleavage has not been fully characterized [19,20]. Therefore we decided to characterize BACE1-mediated cleavage of contactin-2 in cellular and neuronal models and explore whether BACE1 cleavage regulates surface expression of contactin-2, potentially affecting the cell adhesion function of the protein.

## Materials and methods

### Antibodies and reagents

Anti-human contactin-2 (MAB-1714) and anti-mouse contactin-2 (AF4439) antibodies were purchased from R&D Systems (Minneapolis, USA). The rabbit monoclonal anti-human contactin-2 antibody was from Abcam (Cambridge, USA) while rabbit monoclonal BACE1 antibody was from Cell Signaling Inc. (Boston, USA). Anti-mouse V5 antibody was from Life Technologies (Grand Island, USA). Rabbit anti-APP was described earlier [27]. sAPP (22C11), N-cadherin and NrCAM antibodies were from EMD Millipore (Billerica, USA). BACE1 Inhibitor IV was also purchased from EMD Millipore.

### Plasmid construction

Human contactin-2 plasmid (MGC:157722) was obtained from Harvard Plasmid DNA Resource Core (Harvard Medical School, Boston). Contactin-2 cDNA coding region was amplified using the following primers: forward, CACCATGGGGACAGCCACCAGG AGG; reverse, TCAGAGCTCCAGGGAGCCTATGAGG. The amplified fragments were subcloned into pcDNA6.1 vector (directed TOPO system, Life Technologies). For generation of the soluble form of contactin-2, a V5-tag was C-terminally added to inactivate the GPI-anchor domain. The putative BACE1 cleavage site was mutated in the contactin-2 cDNA construct (CNTN2-MM1008AA) with the help of Quick Change site-directed mutagenesis kit from Agilent Technologies (Santa Clara, USA) according to the manufacturer’s protocol using the following primers: forward, GAGGAATGGAGGCACAAGCGCGGCGGTGGAGAACATGGCAGTC; reverse, GACTGCCATGTTCTCCACCGCCGCGCTTGTGCCTCCATTCCTC. All the constructs were sequenced and verified at the MGH DNA sequencing core facility (Boston, USA).

### Generation of contactin-2 stable cell lines

Single cell stable lines were generated for both GPI-anchored contactin-2 and soluble contactin-2 (V5-tagged) in CHO cells. Briefly, 4 μg of GPI-anchored contactin-2 and soluble contactin-2 cDNA were transfected in CHO cells with the help of Effectene transfection reagent (Qiagen, Valencia, USA) according to the manufacture’s protocol. 48 h after transfection, cells were trypsinized and replated in the presence of 10 μg/μl of Blasticidin selection marker. After 2 weeks of selection, Blasticidin resistant cells were further plated in serial dilutions in a 96-well plate in order to get single cell colonies. Later, different single cell clones were picked and analyzed by Western blotting for the optimal expression of contactin-2 with the help of specific antibodies.

### Primary neuronal cultures

Primary neuronal cultures were prepared from 16-day old pregnant female mice (CD-1). Mice were purchased from Charles River Laboratories, Cambridge, and the animal protocol was approved by the MGH Institutional Animal Committee. In brief, hippocampi and frontal cortices were dissected and isolated from pups at embryonic day 16 (E-16). Dissected tissue was further triturated using fine pasture pipette and later plated on poly-D-lysine/laminin coated 6-well tissue culture plate using Neurobasal media containing 2% B-27 serum supplement (Life Technologies). Cultures were maintained in a humidified environment at 37°C with 5% CO_2_ and 50% of the media were replaced every 3^rd^ day.

### Lentiviral generation and infection

BACE1 and the control mCherry lentiviral particles were generated at the MGH Vector Core facility (Charlestown, USA). Contactin-2 expressing CHO cells at 40% confluency were infected with 1 × 10^6^ lentiviral particles. 24 h after infection, media was replaced and the cells were allowed to grow for a total of 5 days before extraction. In case of primary neurons, cultures were infected at DIV5 with 1 × 10^6^ lentiviral particles. In order to reduce the lentivirus-mediated toxicity, 50% of the culture media was replaced 12 h after infection. Cultures were allowed to grow for 6 additional days after infection.

### BACE1 inhibitor treatment

CHO cells stably expressing GPI-anchored contactin-2 were plated on 60 mm tissue culture plate and treated with either 4 μM of BACE1 Inhibitor IV (EMD Millipore) or the same volume of DMSO control vehicles. The treated cells were allowed to grow for 48 h and the media was replaced with fresh media containing BACE1 Inhibitor IV. 48 h after conditioning, both the media and the cells were collected, processed and analyzed by Western blot. For primary neuronal cultures, the neurons were treated with 1 μM BACE1 Inhibitor IV for 48 h, replaced with fresh media containing 1 μM of BACE1 Inhibitor IV, and then incubated for additional 48 h before collecting the media and the cells.

### Western blot analysis

Cells were lysed in 1X GTIP buffer containing 10 mM Tris-HCl (pH 6.8), 2 mM EDTA (pH 8.0), 150 mM NaCl, 1% Triton X-100, 0.25% Nonidet P-40, and a protease inhibitor cocktail (Roche Molecular Biochemicals, Indianapolis, IN, USA). The lysates were centrifuged at 16,000 × g in order to remove insoluble materials and the protein concentration was measured using a BCA protein assay kit (Pierce Biotechnology, Rockford, USA). 25-75 μg protein samples were separated either on 3-8% Tris-Acetate gels, 4-12% gradient Bis-Tris gels, or 12% Bis-Tris gels (Life Technologies) and transferred on PVDF membrane. Blots were then blocked either with 5% skimmed milk or with 5% BSA (Sigma, St. Louis, MO, USA) for overnight at 4°C. Primary antibodies were used at the following dilutions: human contactin-2 (1:200), mouse contactin-2 (1:200), anti-V5 (1:3000), anti-APP C-66 (1:1000), anti-BACE1 (1:1000), anti-sAPPβ (1:200), anti-N-cadherin (1:1000), anti-NrCAM (1:1000) and anti-GAPDH (1:2000). Blots were developed by chemiluminescence using Biomax light film (Kodak, Rochester, USA) or Versa Doc imaging system and quantified using Quantity One software (Biorad).

### Immunofluorescence analysis

CHO cells stably expressing GPI-anchored contactin-2 were plated on glass coverslips in a 6-well tissue culture plate. Cells were treated with 4 μM BACE1 Inhibitor IV or DMSO for 48 h, fixed and then stained with anti-human contactin-2 antibody (1:200) without permeabilization for overnight at 4°C. After incubating with Alexa Fluor488-conjugated secondary antibody, cover slips were mounted on the glass slides with the help of mounting media containing DAPI (Life Technologies). Images were taken on Olympus IX 70 microscope with the same exposure settings and later processed by IPLab software.

### Cell surface biotinylation

Cell surface biotinylation experiments were performed on primary neuronal cultures at day 15 (DIV15). Cultures grown in 6-well plate were washed three times with ice cold Hank’s balanced salt solution (HBSS) and incubated in dark for 1 h with 2 ml of ice cold HBSS containing 0.5 mg/mL Sulfo-NHS-Biotin (Pierce). Five-minute incubation with 100 μM lysine solution was used to quench the reaction followed by three washes with cold HBSS. Cells were then extracted directly in an extraction buffer containing 10 mM Tris-HCl (pH 6.8), 2 mM EDTA (pH 8.0), 150 mM NaCl, 1% Triton X-100, 0.5% Sodium Deoxycolate, 0.2% SDS, 1 mM PMSF, 20 μM ALLN and a protease inhibitor cocktail (Roche Molecular Biochemicals). The insoluble fractions were removed by centrifugation at 16,000 × g and the protein concentrations were determined using the BCA protein assay kit (Pierce). 200-600 μg of protein was immunoprecipitated using NeutrAvidin beads (Pierce) overnight at 4°C. Next day, samples were washed 3 times with the extraction buffer, eluted with the LDS sample loading buffer (Life Technologies) supplemented with 2% (v/v) β-mercaptoethanol and separated on 4-12% Bis Tris-Acetate NuPage gel (Life Technologies) followed by Western blot analysis using various primary antibodies.

### Analysis of AD brain samples

Brain samples from 9 AD and 8 Non-AD patients (age-matched, temporal lobe region) were obtained from Dr. Yong Shen (Roskamp Institute, Sarasota, FL). The same set of samples were previously used to analyze altered sodium channel metabolism [16]. Frozen tissue samples were lysed with extraction buffer containing 10 mM Tris-HCl (pH 6.8), 1 mM EDTA, 150 mM NaCl, 0.25% Nonidet P-40, 1% Triton X-100, 0.2% SDS and a protease inhibitor cocktail (Roche Molecular Biochemicals). 75 μg of protein was resolved on 12% Bis/Tris NuPage gels or 3-8% Tris/Acetate gels. BACE1 levels were determined in our previous study [16] while amyloid plaque density information for individual samples were provided by Dr. Shen’s laboratory.

## Results and discussion

### Contactin-2 is a substrate for BACE1

**Figure 1:**
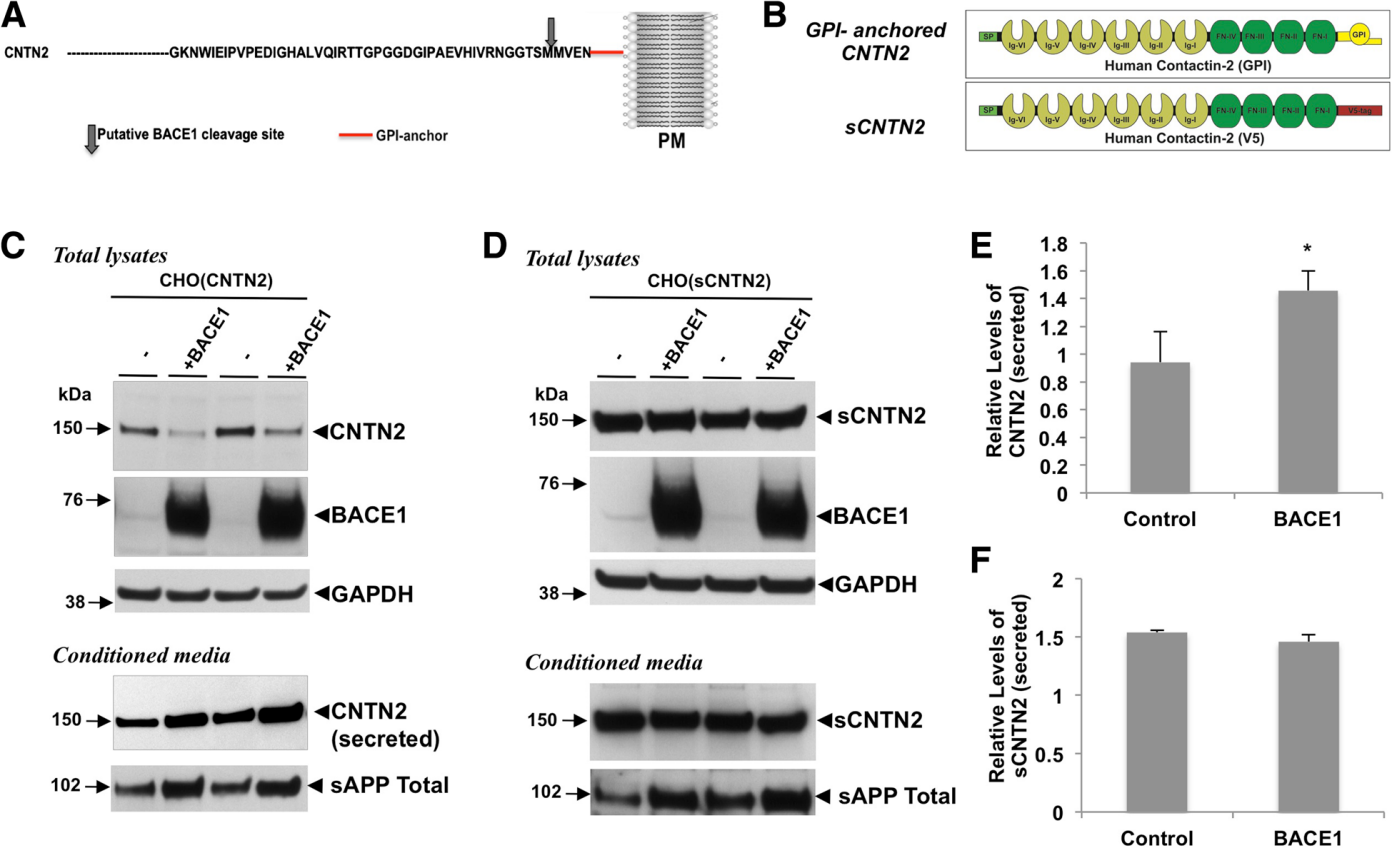
**BACE1 cleaves contactin-2 close to its GPI domain. A)** Schematic representation of partial amino acid sequence of contactin-2 indicating putative BACE1 cleavage site. **B)** Graphic representation of GPI-anchored and soluble contactin-2 proteins that were used in this study. **C)** Lentiviral-mediated overexpression of BACE1 increased contactin-2 and sAPP shedding in CHO cells stably expressing GPI-anchored human contactin-2 (CNTN2). **(D)** Release of soluble contactin-2 (sCNTN2) was not changed by BACE1 overexpression. **E** and **F)** Quantitative analysis of the secreted contactin-2 levels in **C)** and **D)**, respectively (Student t test; *, p < 0.05; n = 3 for each condition).

To investigate the physiological functions mediated by BACE1, we have been identifying novel substrate proteins other than APP [10,16,21]. Toward this goal, we used an unbiased bioinformatics approach and identified a group of candidate substrate proteins that contain putative BACE1 cleavage sites close to the cell membrane. Interestingly, both human and mouse contactin-2 harbor a strong putative BACE1 cleavage site in their extracellular NH_2_-terminal domain 4 amino acids upstream from its GPI membrane linker (Figure 1A). Two previous reports have also suggested that mouse contactin-2 may be a BACE1 substrate [19,20].

To test whether contactin-2 is a BACE1 substrate in cells, we first generated expression constructs with full-length human contactin-2 (Figure 1B, GPI-anchored CNTN2) or secreted contactin-2 where the GPI-anchor domain was inactivated by the addition of a V5-epitope tag (Figure 1B, sCNTN2). These constructs were transfected into Chinese Hamster Ovary (CHO) cells and stable CHO cell clones with high expression of GPI-anchored CNTN2 or sCNTN2, were selected for the experiments (Additional file 1: Figure S1). As expected, Western blot analysis of the conditioned media using a human contactin-2 antibody revealed a strong increase in the secreted form of contactin-2 in CHO cells with sCNTN2 due to the lack of active GPI-anchor domain (Additional file 1: Figure S1A). Interestingly, we also found a small but consistent release of contactin-2 into cell culture media of CHO cells with GPI-anchored contactin-2 (Additional file 1: Figure S1B). To explore whether BACE1-mediated cleavage regulates contactin-2 release into cell culture media, we coexpressed human BACE1 in CHO cells with contactin-2 and analyzed contactin-2 levels in total cell lysates and conditioned cell culture media (Figure 1C and D). BACE1 overexpression increased secreted contactin-2 levels by ~2 fold in CHO cells with GPI-anchored contactin-2 (Figure 1C and E). As a negative control, we co-expressed BACE1 in CHO cells expressing sCNTN2 with the inactive GPI anchor domain (Figure 1D and F). As expected, BACE1 expression did not induce significant changes in soluble contactin-2 levels both in the conditioned media and in the total cell lysate (Figure 1D and F). These data confirm that BACE1 cleaves GPI-anchored contactin-2 and therefore regulates the release of contactin-2 ectodomain.

### Endogenous BACE1 activity regulates contactin-2 cleavage

**Figure 2:**
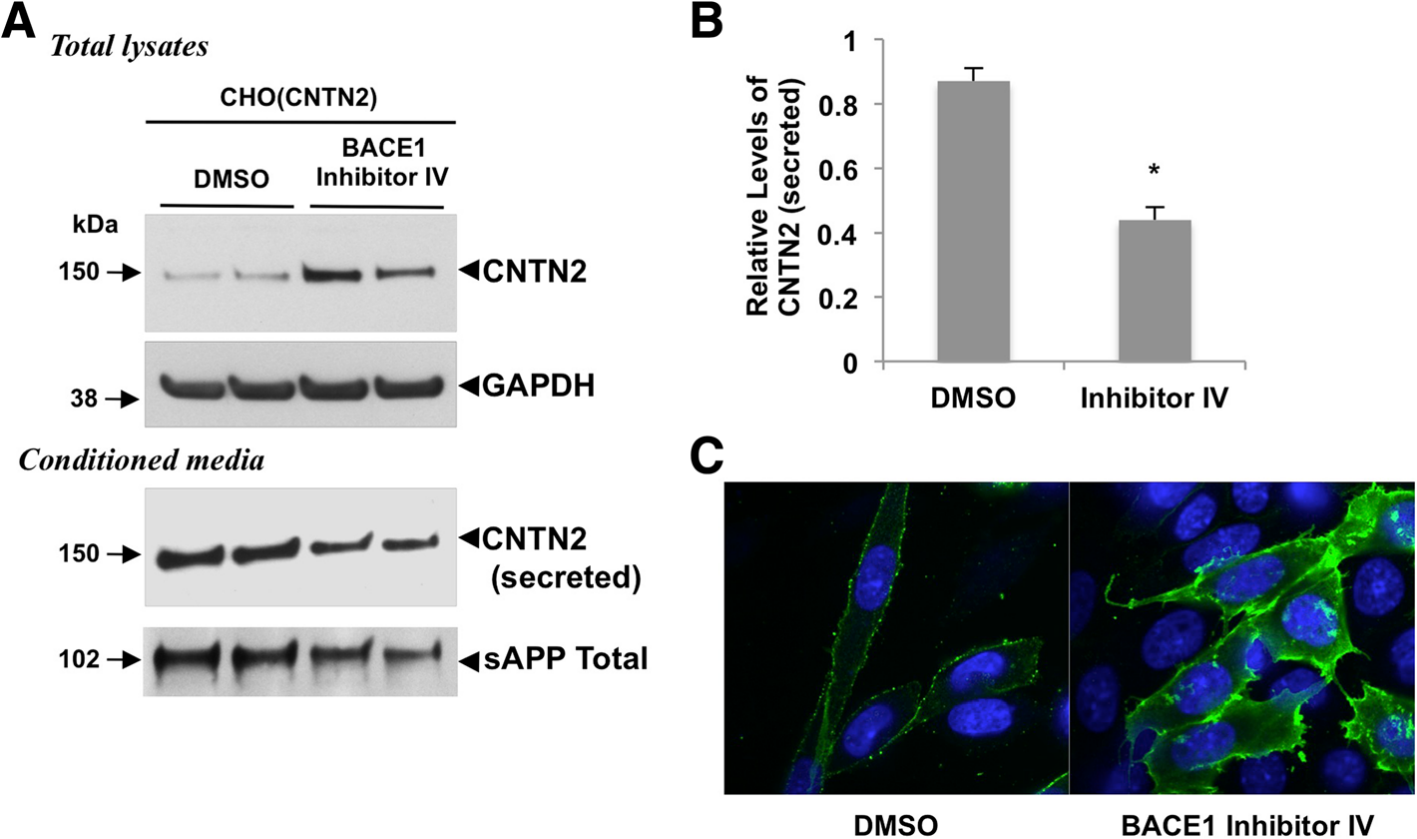
**BACE1 inhibition reduces contactin-2 shedding. A)** Western blot showing a sharp decrease in contactin-2 levels in CHO(CNTN2) cells treated with BACE1 Inhibitor IV. **B)** Quantitative analysis of the secreted contactin-2 levels in **A)** (Student t test; *, p < 0.05; n = 3 for each condition). **C)** Confocal microscopy images of cell surface contactin-2 levels in CHO cells expressing CNTN2. BACE1 Inhibitor IV induced a strong increase in contactin-2 surface levels (green). Nuclear staining with DAPI is shown in blue. Images are taken at identical settings.

As BACE1 enhances GPI-anchored contactin-2 shedding, we next investigated whether contactin-2 cleavage is also regulated by endogenous BACE1 activity. CHO cells expressing GPI-anchored contactin-2 were treated with BACE1 Inhibitor IV for 4 days and changes in contactin-2 levels were assessed by Western blot analysis. As shown in Figure 2A and B, pharmacological inhibition of endogenous BACE1 activity decreased contactin-2 levels by 49% in the conditioned media as compared to the DMSO-treated control cells. We also found that BACE1 Inhibitor treatment dramatically increased total contactin-2 levels in cell lysates (Figure 2A). To confirm that BACE1 Inhibitor treatment blocked endogenous BACE1 activity, we also showed significant reduction in total sAPP levels in the conditioned media (Figure 2A). Finally, contactin-2 cell surface levels were assessed under non-permeabilizing conditions by immunofluorescence analysis with contactin-2 antibody against extracellular domains. As shown in Figure 2C, inhibition of endogenous BACE1 activity by BACE1 Inhibitor IV treatment markedly reduced contactin-2 shedding and thus significant increased contactin-2 cell surface levels as compared to the control one. These data clearly demonstrate that endogenous BACE1 activity regulates contactin-2 cleavage, cell surface contactin-2 levels and release of contactin-2 into the cell culture media.

**Figure 3:**
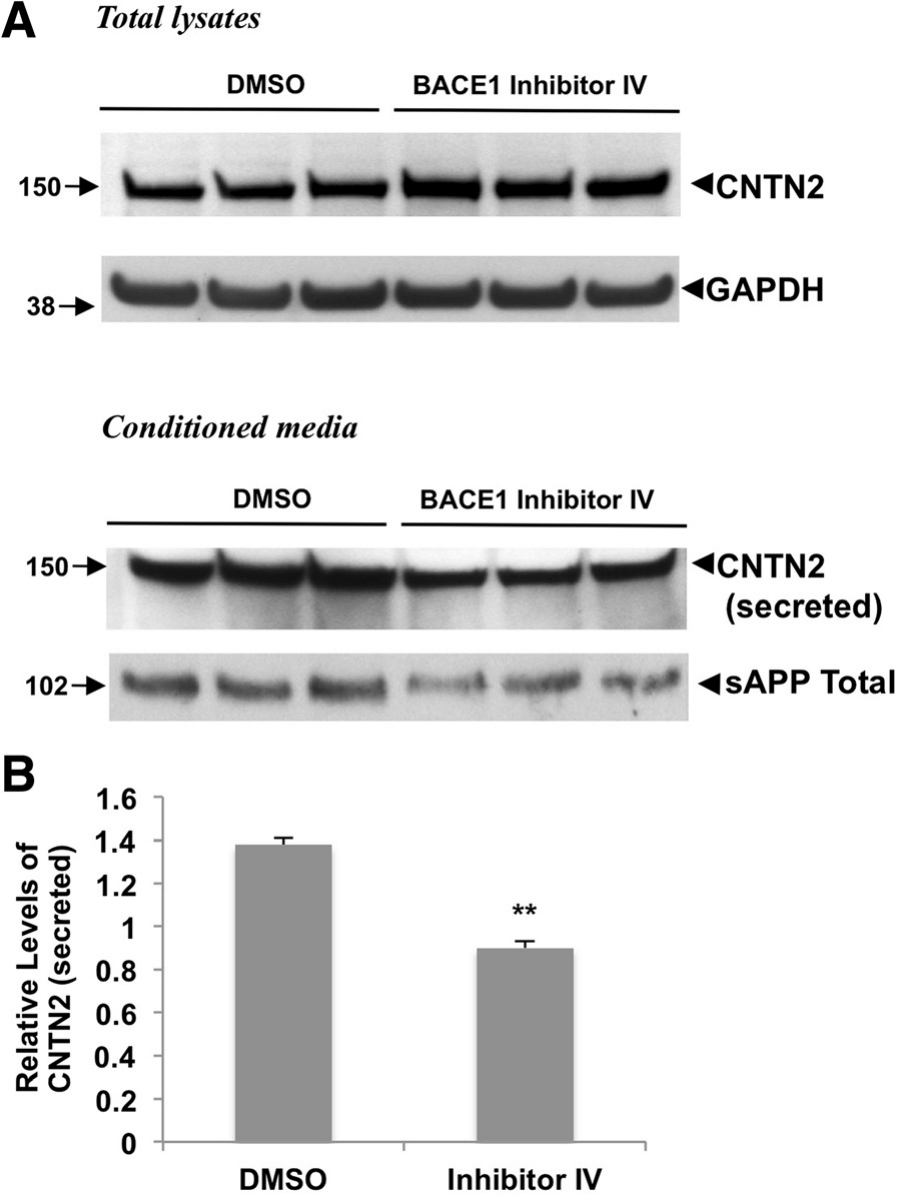
**BACE1 inhibition also decreases release of endogenous neuronal contactin-2. A)** Western blot analysis of mouse primary neuronal cultures treated with 1 μM BACE 1 Inhibitor IV showed a significant decrease in contactin-2 and sAPP levels in the conditioned media. **B)** Quantitative analysis of secreted contactin-2 levels in **A)** (Student t test; **, p < 0.01; n = 3 for each condition).

To test whether BACE1 regulates the cleavage of endogenous contactin-2, we next studied contactin-2 shedding in mouse cortical neuronal cultures. Similarly to CHO cells with GPI-anchored contactin-2, we found that BACE1 inhibitor treatment decreased secreted contactin-2 levels in the conditioned media as compared to DMSO-treated controls (Figure 3A). Quantitation showed an approximately 50% decrease in secreted contactin-2 levels (Figure 3B). Inhibition of endogenous BACE1 activity was also verified by the decreased total sAPP levels as shown in Figure 3B. Our results demonstrate that endogenous contactin-2 is a substrate for BACE1 and undergoes BACE1-dependent cleavage both in CHO cells and primary neuronal cultures.

### BACE1 cleaves contactin-2 at Met1008-Met1009

**Figure 4:**
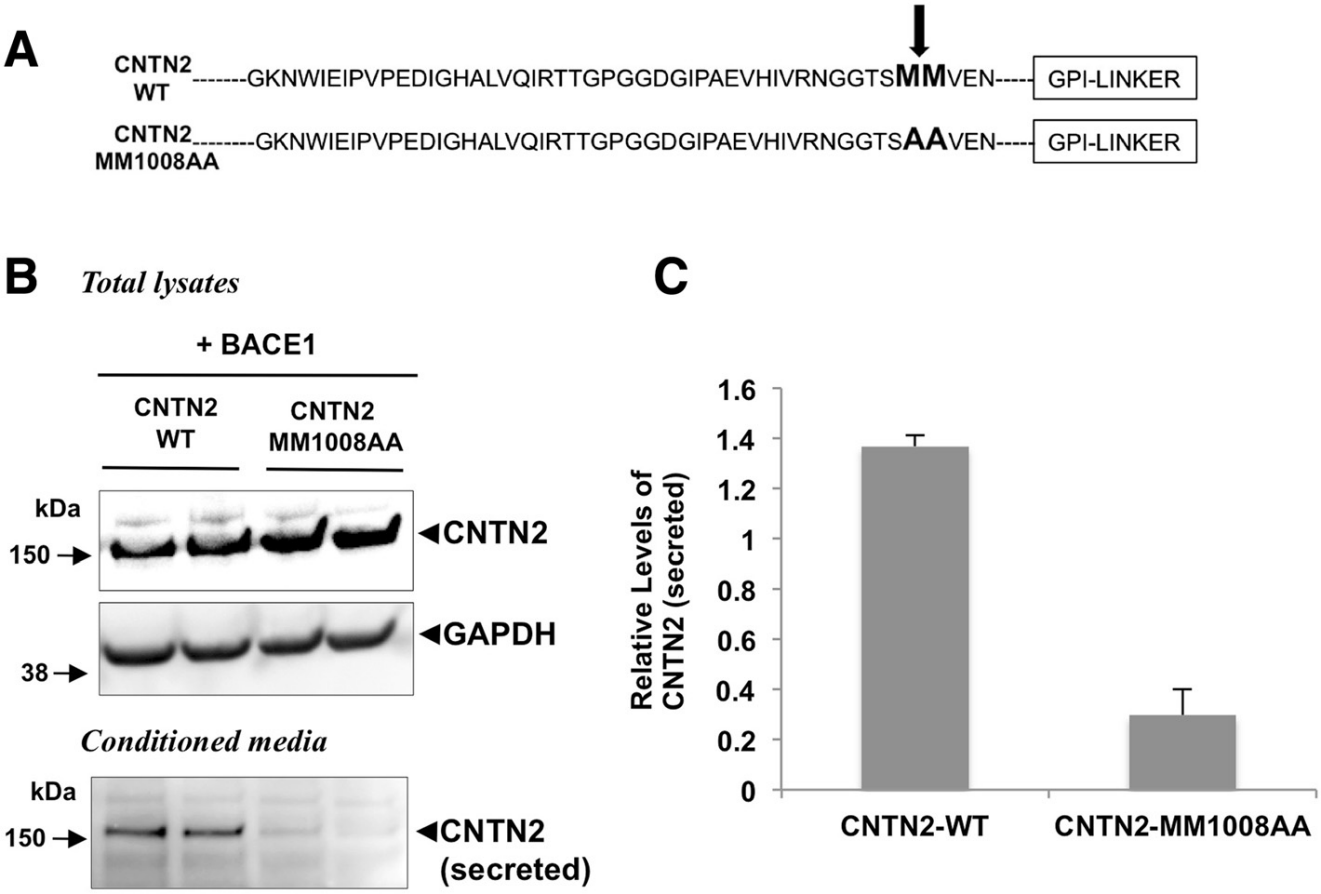
**Site-directed mutagenesis of the putative BACE1 cleavage site specifically blocks contactin-2 shedding. A)** Partial amino acid sequence of human contactin-2 indicating the putative BACE1 cleavage site. CNTN2-MM1008AA mutations were introduced to block BACE1 cleavage. **B)** Western blot analysis showing a dramatic reduction in contactin-2 release in the conditioned media of the cells expressing the BACE1-cleavage site mutant contactin-2 (CNTN2-MM1008AA). **C)** Quantitative analysis of secreted contactin-2 levels in **B)**.

Previously, we have shown that the BACE1 cleavage site mutation 147LM/VI abolished cleavage of the sodium channel β2 subunit [28]. Similarly, we now introduced two mutations, 1008-1009MM/AA, into the putative BACE1 cleavage site in contactin-2 (Figures 1A and 4A) and tested whether these mutations specifically block contactin-2 shedding. To investigate whether the 1008-1009MM/AA mutations affect BACE1-mediated contactin-2 cleavage, we generated CHO cells co-expressing BACE1 and wild-type contactin-2 (CNTN2-WT) or mutant contactin-2 (CNTN2-MM1008AA) and analyzed secreted contactin-2 levels in the conditioned media. We found that soluble contactin-2 levels were significantly decreased in CHO cells expressing CNTN2-MM1008AA as compared to CNTN2-WT control cells (Figure 4B). Quantitative analysis showed that soluble contactin-2 levels were decreased by 78% in CNTN2-MM1008AA cells as compared to wild type control cells (Figure 4C). Together, our data indicate that BACE1-mediated contactin-2 cleavage site is at 1008Met-1009Met and that this cleavage plays a major role in regulating contactin-2 release in cells.

### BACE1 regulates cell surface contactin-2 levels in mouse primary neurons

**Figure 5:**
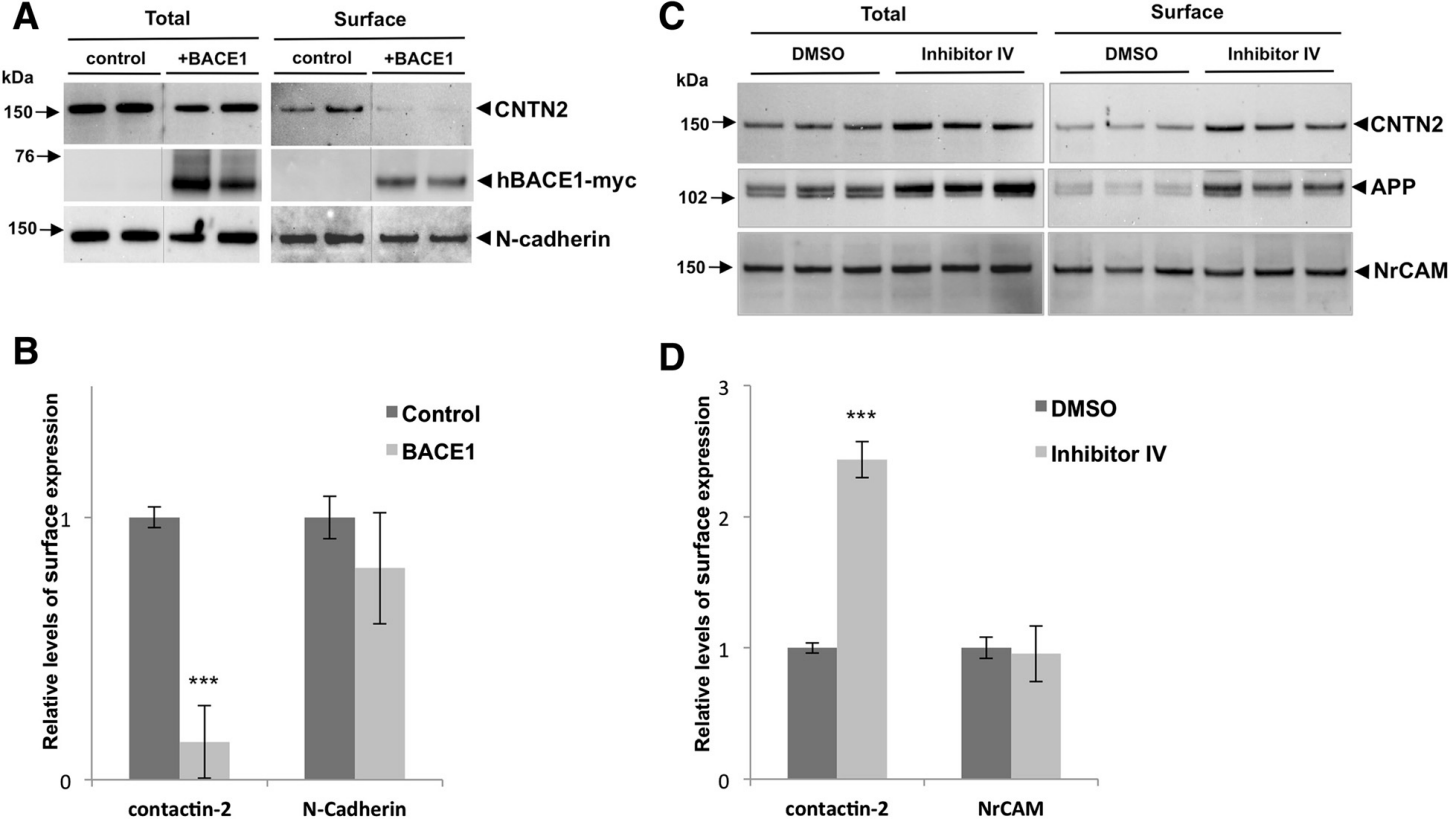
**BACE1 regulates surface levels of contactin-2 in mouse primary neuronal cultures. A)** Western blot analysis showed that surface levels of contactin-2 were largely decreased by BACE1 overexpression in mouse primary neuronal cultures. **B)** Quantitative analysis of surface contactin-2 and the control N-cadherin levels in **A)** (Student t test; ***, p < 0.005; n = 3 for each condition). **C)** Western blot analysis showed that surface levels of contactin-2 were largely increased by BACE1 Inhibitor IV treatments. **D)** Quantitative analysis of surface contactin-2 and the control NrCAM levels in **C)**. (Student t test; ***, p < 0.005; n = 3 for each condition).

We next used cell surface biotinylation of primary mouse neurons to ask whether BACE1 also regulates surface expression of contactin-2 under physiological conditions, in addition to its shedding. To increase BACE1 activity, we infected primary neurons with human BACE1 lentiviral vectors and incubated the cultures for 6 days. BACE1 overexpression dramatically decreased cell surface levels of contactin-2 while it did not significantly affect surface levels of N-cadherin, a type I membrane protein that is not cleaved by BACE1 (Figure 5A and B). Conversely, when mouse primary neurons were treated with BACE1 Inhibitor IV and subjected to surface biotinylation, we observed a strong increase in contactin-2 cell surface levels (Figure 5C and D). Surface levels of APP were concomitantly increased in these neurons (Figure 5C). All together, these data show that BACE1 activity modulates contactin-2 cleavage and thus regulates its surface levels in mouse primary neurons.

### Decreased contactin-2 levels in AD brain samples

**Figure 6:**
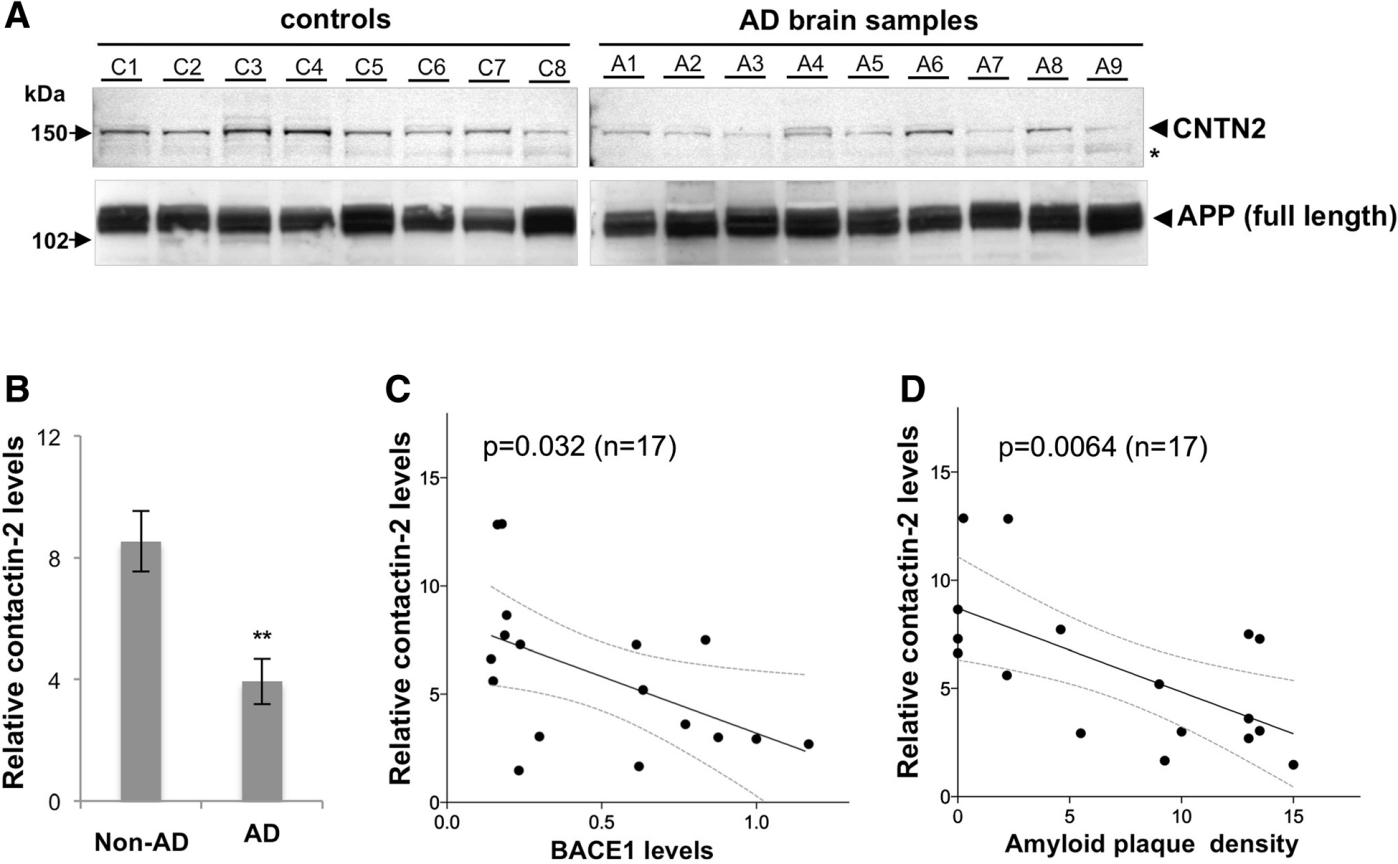
**Contactin-2 levels are decreased in AD brain samples inversely correlating with BACE1 levels or β-amyloid plaques. A)** Western blot analysis showed that contactin-2 levels were decreased in AD brain samples as compared to age-matched controls. **B)** Quantitative analysis of contactin-2 levels in **A)** (**, p < 0.01, Student’s t test; n = 9 for AD, 8 for non-AD). Contactin-2 levels were inversely correlated with BACE1 levels (**C**, p = 0.032, Person’s correlation test; n = 17) or amyloid plaque densities (**D**, p = 0.0064) in these samples.

Studies have shown that BACE1 activity is significantly increased in late-onset AD brains [29-31]. Previously, we reported that cleavage of the voltage-gated sodium channel β2 subunit was increased in AD brain samples, closely correlating with the elevated BACE1 levels [16]. Therefore, we next asked whether contactin-2 cleavage was also increased in the same AD brain sets with elevated BACE1 levels. Unlike APP or the sodium channel β2 subunit, contactin-2 is anchored to the membrane via a GPI domain. Thus, its shedding cannot be assessed by analyzing the levels of its membrane-anchored C-terminal fragments. Instead, we tested whether total levels of contactin-2 are decreased in our AD samples similarly to BACE1 overexpression in our cellular models (Figures 1 and 5). Western blot analysis revealed that contactin-2 levels were generally decreased in AD brain samples as compared to control age-matched samples (Figure 6A). Quantitative analysis showed a significant ~50% decrease in contactin-2 in AD brain samples (Figure 6B; p < 0.01; n = 9 for AD, 8 for non-AD). More importantly, decreased contactin-2 levels were closely correlated with BACE1 levels (Figure 6C; p = 0.032; n = 17) and even more closely with amyloid plaque density (Figure 6D; p = 0.0064; n = 17). The close correlation of contactin-2 levels with amyloid plaque density may also stem from amyloid-induced elevated presynaptic BACE1 in AD brains [32]. Together, our results indicate that elevated BACE1 activity increases contactin-2 cleavage in human brains with high BACE1 levels.

In this study, we showed that BACE1 activity tightly regulates cell surface contactin-2 levels in CHO cells and cultured mouse primary neurons by selectively cleaving cell surface contactin-2. Recent studies indicated that BACE1-null neurons display axon guidance defects but the underlying molecular mechanisms are not fully elucidated [12-14]. While the biological activity of the released contactin-2 remains unknown, lack of proper BACE1-mediated cleavage of surface neural adhesion molecules such as contactin-2 may provide an explanation for axon guidance defects observed in BACE1-null mice [13,20]. Since contactin-2 regulates axon guidance through homophilic and heterophilic interactions with other neural adhesion molecules [25,26,33-38], abnormal accumulation of surface contactin-2 by BACE1 knock-down may interfere the proper axon guidance *in vivo*. In mice, premature early overexpression of contactin-1 (F3/contactin) leads to the reduction in the cerebellar size, granule cell numbers and Purkinje cell maturation [39], which suggests the importance of the precise contactin expression in early brain development. Similarly, Hitt *et al*. recently proposed that decreased BACE1-mediated shedding of CHL1 may also contribute to axon guidance deficits in BACE1-null neurons [13]. Our findings, together with those of Hitt *et al.,* suggest that lack of proper BACE1-mediated shedding of neural adhesion molecules may produce the final phenotype of axon guidance deficits found in BACE1-null neurons. It will be interesting to identify all major neural adhesion molecules mediating the effect of BACE1 on axonal guidance in neural tissues.

Our data in Figure 6 also suggest that elevated BACE1 in brains of AD patients may also contribute to AD pathogenesis by decreasing contactin-2 levels and possibly its surface expression. Mice lacking contactin-2 show deficits in neuronal migration (for a subset of cerebella neurons) [40], neurogenesis [41], learning and memory [26] and ion channel clustering [26,42]. Therefore, decreased contactin-2 levels in brains of AD patients may contribute the neuronal deficits through multiple mechanisms. However, further studies will be required to fully characterize the functional consequences of BACE1-mediated contactin-2 cleavage in AD pathogenesis as well as its potential side-effects during BACE1 inhibitor therapies currently in clinical trials.

While we were investigating human contactin-2 cleavage in our cellular model systems, two unbiased secretome analyses have been published showing that TAG-1 (mouse contactin-2) ectodomain release is regulated by BACE1 activity in mouse primary neuronal cultures [19,20]. Kuhn *et al.* also confirmed that TAG-1 ectodomain release was significantly decreased in brains of BACE1-null mice [19]. Consistent with these findings, we confirmed that BACE1 activity tightly regulates the release of contactin-2 in mouse primary neuronal cultures (Figure 3). Moreover, we characterized human contactin-2 cleavage by BACE1 and identified the cleavage site for the first time (Figures 1, 2 and 4). Our surface biotinylation studies also demonstrated that BACE1 activity tightly regulates cell surface contactin-2 levels in cultured mouse primary neurons (Figure 5). Together, our data show that human and mouse contactin-2 are endogenous substrates for BACE1 and that BACE1-mediated cleavage modulates the surface expression of contactin-2.

## Competing interests

The authors declare that they have no competing interests.

## Authors’ contributions

DMK and DYK designed the study and contributed to the analysis of data. VG, CD, MH, and DYK performed the experiments and collected data. VG, DMK and DYK wrote and revised the manuscript. All authors read and approved the final manuscript.

## Supplementary Material

Additional file 1: Figure S1 Expression of sCNTN2 and GPI-anchored CNTN2 in CHO cells. Western blot analysis showed the overexpressed sCNTN2 is mostly secreted into the conditioned media (A) while GPI-anchored CNTN2 is mostly in the total lysate fraction (B). Click here for file
