# Supplementary material for: BACE1 activity regulates cell surface contactin-2 levels
Source: Mol Neurodegener. 2014 Jan 9;9:4. doi: 10.1186/1750-1326-9-4 (PMC3899608; doi:10.1186/1750-1326-9-4)
Supplement: Additional file 1: Figure S1 — Expression of sCNTN2 and GPI-anchored CNTN2 in CHO cells. Western blot analysis showed the overexpressed sCNTN2 is mostly secreted into the conditioned media (A) while GPI-anchored CNTN2 is mostly in the total lysate fraction (B). [file 1750-1326-9-4-S1.pdf]

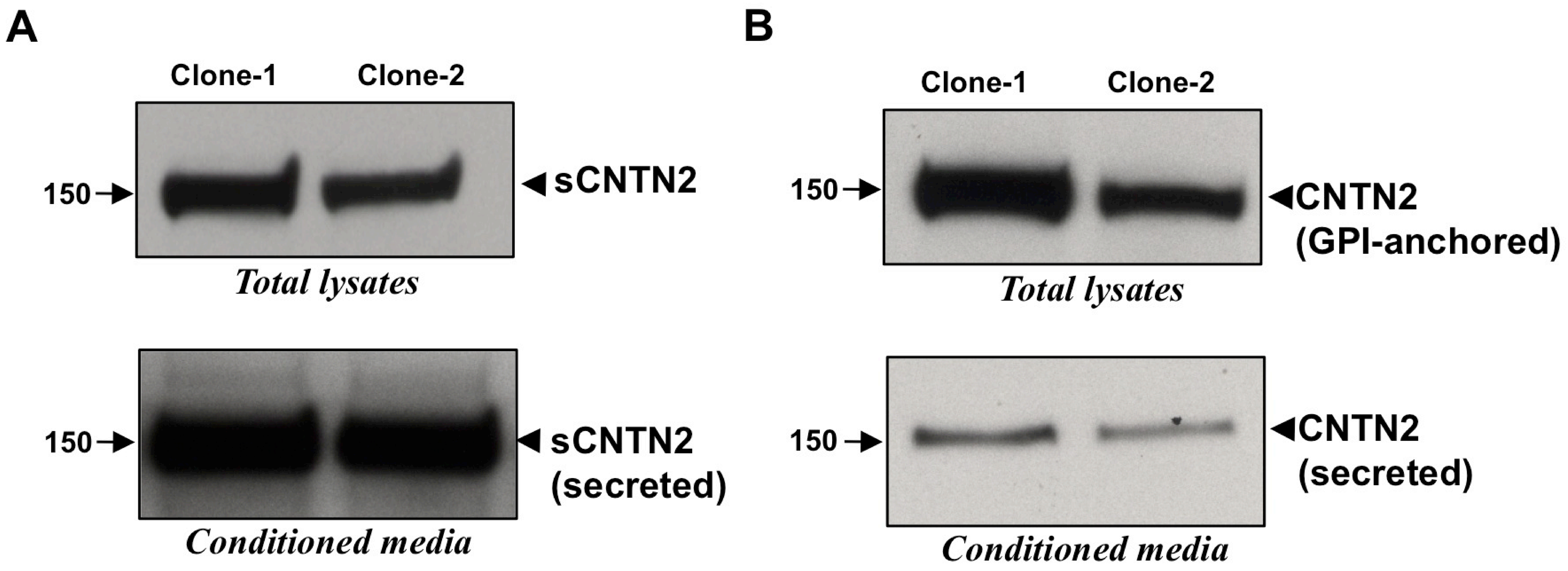

Supplemental Fig. 1

Supplemental Figure Legends:

Supplemental Figure 1. Expression of sCNTN2 and GPI-anchored CNTN2 in CHO cells

Western blot analysis showed the overexpressed sCNTN2 is mostly secreted into the conditioned media (A) while GPI-anchored CNTN2 is mostly in the total lysate fraction (B).
